# Pre-clinical atherosclerosis is found at post-mortem, in the brains of men with HIV

**DOI:** 10.1007/s13365-020-00917-1

**Published:** 2021-01-06

**Authors:** Olusola Daramola, Hebah Ali, Chris-Anne Mckenzie, Colin Smith, Laura A. Benjamin, Tom Solomon

**Affiliations:** 1 Institute of Infection, Veterinary and Ecological Sciences, University of Liverpool, Liverpool, L69 7BE UK; 2 Wirral University Teaching Hospital NHS Foundation Trust, Arrowe Park Wirral, Birkenhead, CH49 5PE UK; 3 Haematological Malignancy Diagnostic Service (HMDS, St James University Hospital Leeds, Leeds Teaching Hospitals NHS Trust, Leeds, LS1 3EX UK; 4 Centre for Clinical Brain Sciences, University of Edinburgh, Edinburgh, EH16 4SB UK; 5 Stroke Research Centre, UCL Queen Square Institute of Neurology, First Floor Russell Square House 10-12 Russell Square, London, WC1B 5EH UK; 6 NIHR Health Protection Research Unit in Emerging and Zoonotic Infections, University of Liverpool, Liverpool, L69 7BE UK; 7 Walton Centre NHS Foundation Trust, Liverpool, L69 7LJ UK

**Keywords:** HIV, Post-mortem, Atherosclerosis, Cerebrovascular disease, ART

## Abstract

The aim of this study is to ascertain the burden of pre-clinical atherosclerotic changes in the brains of young adult males with HIV and explore the impact of anti-retroviral therapy (ART). The study design is case-control, cross-sectional. Histological sections from HIV-positive post-mortem brain samples, with no associated opportunistic infection, from the MRC Edinburgh brain bank were evaluated. These were age and sex matched with HIV-negative controls. Immunohistochemical stains were performed to evaluate characteristics of atherosclerosis. The pathological changes were graded blinded to the HIV status and a second histopathologist reassessed 15%. Univariable models were used for statistical analyses; *p *≤ 0.05 was considered significant. Nineteen HIV-positive post-mortem cases fulfilled our inclusion criteria. Nineteen HIV-negative controls were selected. We assessed mostly small-medium-sized vessels. For inflammation (CD45), 7 (36%) of the HIV+ had moderate/severe changes compared with none for the HIV− group (*p *< 0.001). Moderate/severe increase in smooth muscle remodeling (SMA) was found in 8 (42%) HIV+ and 0 HIV− brains (*p *< 0.001). Moderate/severe lipoprotein deposition (LOX-1) was found in 3 (15%) and 0 HIV−brains (*p *< 0.001). ART was associated with less inflammation [5 (63%) no ART versus 2 (18%) on ART (*p *= 0.028)] but was not associated with reduced lipid deposition or smooth muscle damage. In HIV infection, there are pre-clinical small- to medium-sized vessel atherosclerotic changes and ART may have limited impact on these changes. This could have implications on the increasing burden of cerebrovascular disease in HIV populations and warrants further investigation.

**Electronic supplementary material:**

The online version of this article (10.1007/s13365-020-00917-1) contains supplementary material, which is available to authorized users.

## Introduction

In the era of anti-retroviral therapy (ART), the phenotype of individuals infected with HIV has changed. The combined effect of long-term ART, chronic HIV infection with virological suppression, and advancing age is altering the disease pattern (Gutierrez et al. 2016; Sico et al. 2015; Sacktor et al. 2016; Deeks et al. 2013). For example, stroke and neurocognitive impairment are increasingly more prevalent in treated HIV individuals (Sacktor et al. 2016; Gutierrez et al 2015). The mechanism is unclear and likely multifactorial (Sico et al. 2015). However, accelerated atherosclerotic disease is thought to underlie the increasing burden of cerebrovascular disease, especially in high-income countries (Benjamin et al. 2012). The effects of a dysregulated immune system, endothelial dysfunction, deranged metabolic disturbance and ART toxicity have all been postulated to accelerate atherosclerosis (Deeks et al. 2013; Gutierrez et al. 2015; Benjamin et al. 2012). The association with low nadir CD4 count and cerebrovascular disease also suggests that early biological activities may predispose individuals to future cerebrovascular events (Gutierrez et al. 2016; McArthur and Smith 2013). Studies thus far have relied on soluble biomarkers in the blood and correlated this to surrogate markers of cardiovascular disease such as coronary arterial remodelling (Miller et al. 2015) and carotid intimal media thickness (Hsue et al. 2012). Only a handful of studies have looked at post-mortem brain tissue to explore the mechanism of cerebrovascular disease in HIV infection; two have specifically focused on large vessel remodelling and inflammation in less advanced diseases (Gutierrez et al. 2016, 2015) and one on crude small vessel neuropathological changes in AIDS patients (Connor et al. 2000). To date, no study has considered the specific components that define atherosclerosis (inflammation, lipid deposition and smooth muscle remodelling) in HIV populations and the impact of ART on small- to medium-sized vessels. To bridge this gap, we looked for pre-clinical atherosclerotic changes in HIV-infected post-mortem brain in the early phase of HIV infection and the impact ART has on these changes.

## Methods

The study was carried out at the Centre for Clinical Brain Sciences, University of Edinburgh, and samples were obtained from the Medical Research Council (MRC) Edinburgh brain bank, UK. These included patients who died between 1989 and 2003. Formalin-fixed paraffin-embedded post-mortem brain samples from HIV-positive patients were selected. Those with opportunistic CNS infections or neoplasia were excluded. Age- and sex-matched HIV-negative controls were also selected applying the same exclusion criteria. The causes of death in both cases and controls are listed in Table 1. Ethical approval was sought and approved by the East of Scotland Research Ethics Service (16/ES/0084). For each sample, sections were taken from the frontal convexity and basal ganglia. Haematoxylin and Eosin (H&E) staining was performed to assess the general overview of vascular changes.

**Table 1:** Demographic and clinical characteristics of HIV-positive and negative populations studied

	HIV group (*n* = 19)	Control group (*n* = 19)
Median age, year (IQR)	30 (28–34)	30 (25–37)
Duration of HIV infection median years (IQR)	6(3.5–8.75)	-
ART (%)	11 (58%)	**-**
AZT alone	7 (63%)	-
AZT combination	3 (27%)	-
Stavudine	1(9%)	-
Median CD4 count cells/mm^3^	45(18–60)	**-**
**Cause of death**		
Pneumonia	11 (57%)	-
Drug overdose/alcohol	3 (16%)	5 (26%)
Heart disease	1 (5%)	7 (37%)
Others^a^	4 (21%)	7 (37%)

^a^Other; common causes in this category included drowning and trauma *ART* antiretroviral therapy, *AZT* zidovudine

### Histological assessment

All cases were reviewed, and the assessor was blinded to their HIV status. H&E sections were initially assessed for classical characteristics of small vessel disease (arteriolosclerosis and lipohyalinosis).

### Immunohistochemistry

Immunohistochemical stains were performed to evaluate atherosclerotic changes. We looked specifically for smooth muscle damage, inflammation, and lipid deposition. CD31 (1:100 Dako) was used to detect vascular endothelial cells, HIV-1 p24 (1:50 Dako) for detection of HIV infected cells, leukocyte common antigen (CD45) (1:100 Dako) for detection of inflammation (lymphocytes), lectin-like oxidized low-density lipoprotein receptor-1 (LOX-1) (1:50 Abcam) for detection of lipid deposition (lipid laden macrophages), and smooth muscle actin (SMA) (1:500 Dako) for detection of vascular smooth muscle proliferation/disruption/damage. Positive control tissues included tonsil (CD31 and CD45), brain for HIV-1 p24, large vessel atherosclerosis for LOX-1, and large bowel for SMA. The primary antibody was withheld in negative control.

### Grading of vascular changes and size

**Fig. 1:**
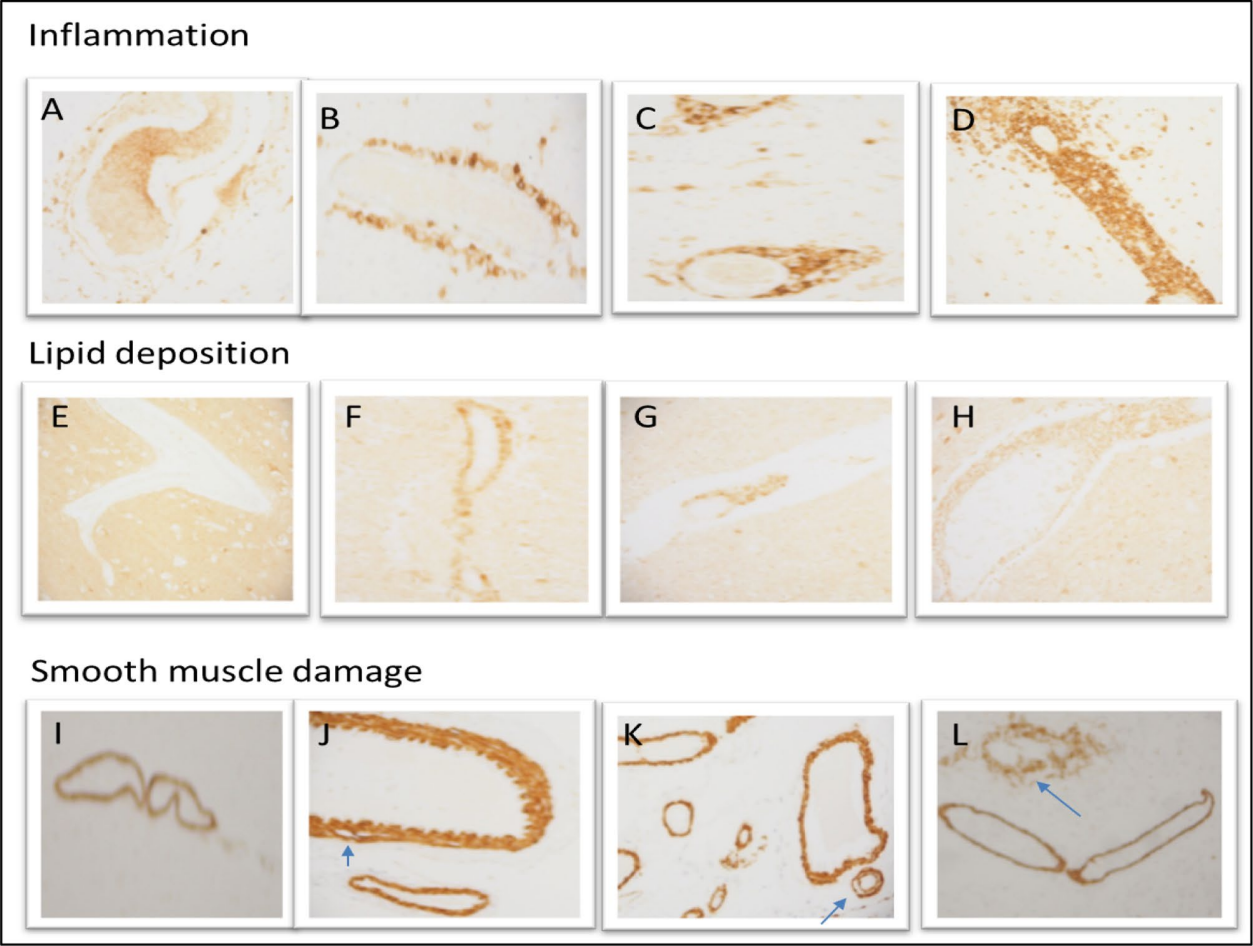
Evidence of pre-clinical atherosclerosis among our HIV population: inflammation—CD45 staining showing perivascular cuffing of lymphocytes **a** normal–less than 4 lymphocytes, **b** mild–linear lymphocytic infiltrates, **c** moderate–partial layering of lymphocytic infiltrates, **d** severe–circumferential layering of infiltrates with obliteration of vascular lumen. Lipid deposition—LOX-1 staining showing lipid-laden macrophages (lipophages) in the vessel wall **e** normal–no lipophage, **f** mild > 6 lipophages, **g** moderate–partial layering of lipophages, **h** severe–circumferential layering of lipophages smooth muscle damage—SMA staining showing disruption of vascular smooth muscle **i** normal–no disruption of muscle fibres, **j** mild–focal separation of muscle fibres, **k** moderate–prominent separation of muscle fibres, **l** severe–disruption of vascular wall with alteration in vessel outline. Magnification × 20

Atherosclerotic changes were graded from normal (no feature of atherosclerosis) through mild (+), moderate (2+) and severe (3+) (Fig. 1) using modification of methods that have been described previously (Fujishiro et al. 2011). Inflammation was graded by the extent of perivascular cuffing of lymphocytes; lipid deposition was graded according to the increasing number of lipid-laden macrophages. Smooth muscle damage was evaluated based on severity of degenerative changes and mal-alignment (disruption) of smooth muscle fibres. The arterial vessels were grouped according to their size: small vessels (diameter = 100–300 µm), small- to medium-sized vessels (diameter > 300–1000 µm), medium-sized vessel (> 1000–10,000 µm) and large-sized vessels (diameter > 10,000 µm).

### Analysis

Grading of each slide was performed 3 times with an average taken. A second histopathologist assessed reliability of the grading technique by random selection of 15% of the total samples. Weighted kappa statistics were used to determine the level of agreement. Comparisons were made with a post-mortem specimen of the coronary artery of an elderly man with severe atherosclerosis, who died of a haemorrhagic stroke; this was done to confirm that the characteristics of established atherosclerosis were consistent with those seen in the HIV population.

Results were presented as proportions (classified as normal/mild/moderate/severe). Quantitative variables were expressed as medians with interquartile ranges. Fisher exact/Chi-squared test was used to compare categorical data and Mann-Whitney *U* for continuous variable. A *p* value of ≤ 0.05 was taken as significant.

## Results

### Description of the cohort

From 168 HIV-positive post-mortems, we identified 19 men without opportunistic infections. The median age was 30 (IQR = 28–34), and the median CD4+ count was 45 (IQR = 18–60). The characteristics of the cohort are reported in Table 1. The validation of grading process showed 97% agreement between two independent observers. The overall weighted kappa (K) was 0.80 (Std. Err ± 0.16, *p* < 0.0001).

CD31 immunolabelling detected endothelial cells in both case and control groups. HIV infection was confirmed by evidence of positive HIV-1 P24 antibody in 18/19 (95%) HIV-positive cases. The only case negative for HIV-1 P24 had diagnosis of HIV at autopsy with very low viral count, indicating recent seroconversion.

## Evidence of pre-clinical atherosclerosis

This study focused predominantly on small-medium-sized vessels of the basal ganglia and frontal convexity, both of which demonstrated similar features. There was no evidence of classical small vessel disease pathology, and no lacunar infarcts were seen. Similar features (Fig. 1) show the spectrum of pre-clinical atherosclerosis found in our HIV population.

When looking specifically at inflammation, there were 9 (47%) mild, 5 (26%) moderate and 2 (10.5%) severe cases of perivascular cuffing of lymphocytes in the HIV-positive group whilst in the HIV-negative group, 2 (10.5%) were mild, 17 (89.5%) were normal, and there were no moderate or severe changes (*p* value < 0.001).

For lipid deposition, 15 (79%) cases were mild, 1 (5%) moderate and 2 (10.5%) severe in the HIV-positive group. There was no evidence of lipid deposition in the control group (*p* value < 0.001).

For vascular smooth muscle damage, there were 9 (47%) mild, 6 (32%) moderate and 2 (10.5%) severe cases, whilst for the control group, 10 (53%) were mild and 9 (47%) were normal; there were no moderate or severe changes in the control group (*p* value < 0.001).

## The effect of anti-retroviral therapy (ART)

Being on ART was associated with less inflammation [5 (63%) no ART versus 2 (18%) on ART (*p* = 0.028)]. There was no difference seen between ART and non-ART cases regarding lipid deposition and vascular smooth muscle damage (Supplement Table 1).

## Comparison with classical changes of atherosclerosis

Changes identified in the control case with classical atherosclerosis were consistent with those seen in the HIV-positive group (Supplement Figure 1).

## Discussion

Our study, of adult males with advanced HIV infection, showed significant evidence of perivascular inflammation, lipid deposition and vascular smooth muscle damage, in small-to-medium-sized vessels when compared with controls of similar age and sex. Treatment of HIV infection was associated with a lower degree of perivascular inflammation but had no significant effect on lipid deposition and vascular smooth muscle damage.

Previous studies have shown that in HIV infection, intracranial arterial remodelling including intimal thickening and media thinning is common in large arteries when compared with an age- and sex-matched HIV-negative cohort and occurs in the context of inflammation (Gutierrez et al. 2016; Gutierrez et al. 2015). We expand the spectrum by demonstrating pre-clinical atherosclerotic changes in small-to-medium-sized arterial vessels, and in addition to inflammation, we observed lipid deposition and smooth muscle damage. Histologically, these changes were not typical of either arteriolosclerosis or lipohyalinosis but suggest accelerated small vessel atherosclerosis. We did not corroborate the findings with large arterial vessel disease, largely due to the choice of sections used for our analysis. Despite this, pre-clinical atherosclerotic changes are common in younger individuals at a relatively early stage of their HIV infection and could predispose HIV-infected individuals to stroke and neurocognitive morbidity. Notably, a radiological review of 60+ HIV-positive stroke patients had a significantly higher number of basal ganglia stroke subtype when compared with an HIV-negative group. Because basal ganglia stroke is largely caused by disease of small-to-medium-sized arteries, this observation is consistent with our findings (Benjamin et al. 2017).

Whilst perivascular inflammation may be reduced by ART use, it may not arrest the remodelling process (i.e. smooth muscle damage and lipid deposition), as suggested from our results. Although our numbers are too small to be definitive, our observation is consistent with the wider epidemiological view that the prevalence of stroke and cognitive impairment remains higher than the respective HIV-negative population, despite the use of ART (Sacktor et al. 2016; Ovbiagele and Nath 2011). Persistent immune activation, metabolic dysregulation due to HIV or ART itself and ART toxicity are all potential mechanisms that have been postulated (Deeks et al. 2013; Gutierrez et al. 2015; Benjamin et al. 2012). The latter could be relevant given the use of drugs like Stavudine during this era. However, Stavudine use was uncommon in our cohort.

What remains unclear is whether events at the early phase of HIV infection predispose individuals to future cerebrovascular disease. The historic association with a low nadir CD4+ count, among those with stable HIV infection, and cerebrovascular risk, is of course, compelling in this debate (Gutierrez et al. 2015; McArthur and Smith 2013). However, the recent introduction of ART in asymptomatic patients (Lundgren et al. 2015) may address this issue. Importantly, as our study suggests, ART may have very little impact on vascular atherosclerotic changes, and therefore, a more definitive evaluation with a larger autopsy cohort would be a critical step forward in clarifying this issue.

This study does have some limitations. Although the sample size was small, this pilot work highlights observations at a biological level that are consistent with epidemiological observations. We were unable to determine the prevalence of vascular risk factors such as smoking, hypertension and diabetes in our population, and it is plausible that these unmeasured factors could have confounded our results. However, in an independent study of a similar population, these factors were uncommon, so unlikely to have affected our findings (Connor et al. 2000). These findings are only relevant to men. The availability of post-mortem sections of the brain among individuals with HIV infection is limited. We therefore relied on a historical cohort of individuals who died between 1989 and 2003 when ART was limited to zidovudine and/or a nucleoside reverse transcriptase inhibitor (NRTIs). Although there is some overlap with current combination therapies, generalisation of our findings will have to be taken in the context of this limitation. However, a recent study exploring the early initiation of ART versus delayed ART, in asymptomatic HIV infection, showed no benefit in preventing cardiovascular disease, among the early initiators of ART; this is consistent with our findings (Lundgren et al. 2015).

In conclusion, preclinical atherosclerotic changes of small-to-medium-sized intracranial arteries are seen within the first 6 years of HIV infection. ART may reduce inflammation but does not appear to impact on arterial remodelling. Further research should focus on clarifying the role of ART on intracranial arterial remodelling.

## Electronic supplementary material

Below is the link to the electronic supplementary material.

Supplementary file1 (DOCX 12.4 kb)

Supplementary file2 (DOCX 549 kb)
