# Supplementary material for: Pre-clinical atherosclerosis is found at post-mortem, in the brains of men with HIV
Source: J Neurovirol. 2021 Jan 6;27(1):80–5. doi: 10.1007/s13365-020-00917-1 (PMC7921050; doi:10.1007/s13365-020-00917-1)
Supplement: Supplementary file 1 — Supplementary file1 (DOCX 12.4 kb) [file 13365_2020_917_MOESM1_ESM.docx]

**Supplement Table I: Pre-clinical atherosclerosis and ART use**

| Moderate/severe | ART negative n=8 | On ART n=11 | P value |
| --- | --- | --- | --- |
|  |  |  |  |
| Inflammation | 5(63%) | 2 (18%) | 0.028 |
| Lipid deposition | 1 (12.5%) | 2 (18%) | 0.737 |
| Smooth muscle damage | 3(37.5%) | 5 (45%) | 0.728 |
