# Supplementary material for: Pre-clinical atherosclerosis is found at post-mortem, in the brains of men with HIV
Source: J Neurovirol. 2021 Jan 6;27(1):80–5. doi: 10.1007/s13365-020-00917-1 (PMC7921050; doi:10.1007/s13365-020-00917-1)
Supplement: Supplementary file 2 — Supplementary file2 (DOCX 549 kb) [file 13365_2020_917_MOESM2_ESM.docx]

**Classical atherosclerosis in a HIV**

**negative case**


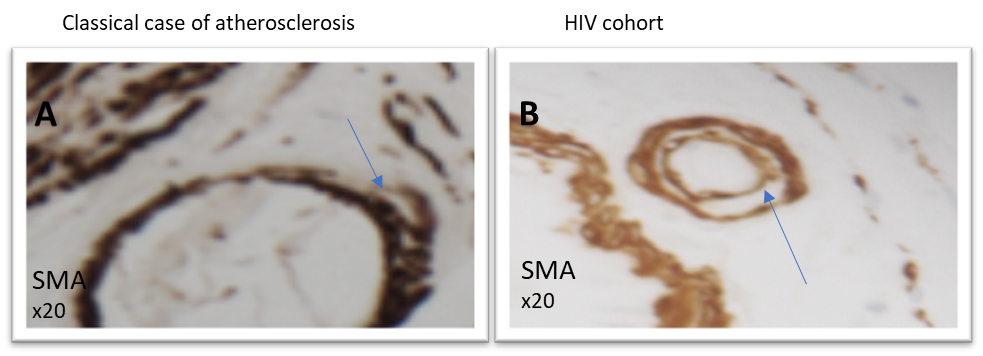


**Supplement Figure I: Comparison of a classical atherosclerotic features with those found in HIV infection**

(A) SMA stain of coronary artery of an elderly gentleman with classical atherosclerosis showing disruption of smooth muscle fibres (B) SMA stain of cerebral vessel in an HIV+ individual showing similar changes
